# Ferrate (VI) Oxidation Is an Effective and Safe Way to Degrade Residual Colistin - a Last Resort Antibiotic - in Wastewater

**DOI:** 10.3389/fvets.2021.773089

**Published:** 2021-12-24

**Authors:** Liqi Wang, Shiming Lv, Xiaoying Wang, Baosheng Liu, Zhong Wang

**Affiliations:** ^1^Laboratory of Animal Genetics, Breeding and Reproduction in the Plateau Mountainous Region, Ministry of Education, Guizhou University, Guiyang, China; ^2^College of Animal Science and Technology, Jiangxi Agricultural University, Nanchang, China

**Keywords:** degradation, colistin sulfate, humic acid, antibacterial activity, toxicity, water

## Abstract

The rise of novel *mcr* mobile resistance genes seriously threatens the use of colistin as a last resort antibiotic for treatment of multidrug-resistant Gram-negative bacterial infections in humans. Large quantities of colistin are released annually into the environment through animal feces. This leads to environmental toxicity and promotes horizontal transmission of the *mcr* gene in aqueous environments. We examined colistin degradation catalyzed by the presence of strong oxidant Fe (VI). We found almost complete colistin degradation (>95%) by Fe (VI) at initial colistin levels of 30 μM at a molar ratio of Fe (VI): colistin of 30 using an initial pH 7.0 at 25°C for 60 min. The presence of humic acid did not alter the degradation rate and had no significant impact on the removal of colistin by Fe (VI). Quantitative microbiological assays of Fe (VI)-treated colistin solutions using *Escherichia coli, Staphylococcus aureus*, and *Bacillus subtilis* indicated that the residual antibacterial activity was effectively eliminated by Fe (VI) oxidation. Luminescent bacteria toxicity tests using *Vibrio fischeri* indicated that both colistin and its degradation products in water were of low toxicity and the products showed decreased toxicity compared to the parent drug. Therefore, Fe (VI) oxidation is a highly effective and environment-friendly strategy to degrade colistin in water.

## Introduction

Veterinary antibiotics are routinely used globally to control infectious diseases and to improve animal growth and feed efficiency (1). However, a large proportion of administered antibiotics are excreted in urine and feces as parent compounds due to poor gut absorption or incomplete metabolism (2, 3). China produces and consumes the most antibiotics of any country and 58% of the annual accumulation of 84,000 tons is excreted by animals (4, 5). This continued release into the environment combined with incomplete removal during wastewater treatment has resulted in the presence of these veterinary antibiotics in aquatic environments (6, 7). Antibiotic residues in water can alter ecosystems and even lead to the development of antimicrobial resistance and this is one of the most serious global threats to human and animal health (8–10). Therefore, measures to degrade or remove antibiotics during wastewater treatment are essential to reduce their harm to the environment, animals and humans.

Colistin (also known as polymyxin E) belongs to the polymyxin family of polypeptide antibiotics derived from *Bacillus polymyxa* var. *colistinus* and was introduced to clinical practice in the late 1950s (11). The bactericidal activity of colistin is essentially that of a cationic detergent that associates with Gram-negative bacterial cell membrane phospholipids and Lipid A. This association leads to rapid permeability changes in the membrane causing leakage of intracellular components, ultimately resulting in cell death (12). Most Gram-negative bacteria are susceptible to colistin and these include the most prevalent animal and human pathogens such as *Enterobacter aerogenes, Escherichia coli, Haemophilus influenzae, Bordetella pertussis, Salmonella* spp*., Legionella pneumophila, Shigella* spp*., Klebsiella pneumoniae, and Pseudomonas aeruginosa* (12). Colistin is widely used in animal farming and especially poultry and swine production because it is extremely effective for treating Gram-negative intestinal infections caused by *E. coli, Salmonella* spp., and *Pseudomonas* spp. Colistin is also used in Asia, Europe, and North America for animal growth promotion but its neuro- and nephrotoxicity restrict its use in humans (9, 12–14). The emergence of multidrug-resistant Gram-negative bacteria (e.g., *Enterobacteriaceae, P. aeruginosa*, and *Acinetobacter baumannii*) over the past decade has caused resurgence of colistin use as a last resort antibiotic for human infections (15).

When administered orally, colistin is very poorly absorbed by the gastrointestinal tract and is eliminated almost unchanged and thereby reaches water bodies or wastewater treatment facilities in its active form (12, 16). Conventional wastewater treatment methods do not adequately remove colistin. For instance, we have determined the colistin levels in swine wastewater collected from pig farms in central China and the results revealed colistin concentrations ranging from 145 to 10,628 ng L^−1^. A toxicity study on the earthworm *Eisenia fetida* revealed that colistin could up-regulate heat shock protein 70 and inhibit metallothionein gene expression while damaging mitochondria and the endoplasmic reticulum (17). In addition to these direct environmental effects of colistin, the development of resistance is increasing. It was formerly believed that bacterial resistance to polymyxins was very low and primarily the result of mutations in chromosomal genes. However, the discovery of a novel mobile colistin resistance mechanism encoded by *mcr*-1 on a mobilizable plasmid by has altered this notion (9, 18). Nine additional *mcr* genes (*mcr-*2-10) have since been discovered from animals and food isolates as well as human clinical strains (19–24). A wide range of human and animal pathogens have been identified that carry these plasmid-mediated resistance genes and this poses a huge threat to sustaining effectiveness of colistin against clinical infections caused by carbapenemase-producing carbapenem-resistant *Enterobacteriaceae*. This scenario heralds the destruction of the last family of the last resort antibiotics, the polymyxins (9, 18, 25).

Recent assessments of the risk of developing resistance when used as a feed additive has been raised by the European Union in June 2016 and China banned colistin as feed additive in April 2017 (26). Nonetheless, colistin still threatens ecosystems and human health thus efficient treatment strategies are needed to remove colistin from wastewater before environmental discharge.

Ferrate VI (FeO${}_{4}^{2-}$) [Fe (VI)] is an extremely effective oxidant that is in current use for bioremediation of wastewater (27–29). Under acidic conditions, the redox potential of Fe (VI) (2.2 V) is greater than ozone (2.07 V) and Fe (VI) is considered to be the strongest disinfectant and oxidant that are currently in use for wastewater and water treatment (28). This inorganic compound is environmentally friendly and its by-product is the non-toxic Fe (III) (30–32). Fe (VI) is a multipurpose treatment chemical and has been used to coagulant or precipitate arsenic (33) and heavy metals (34) and is also a powerful disinfectant for bacteria (31, 35) and viruses (36). Pre-treatment of an aqueous solution requires only a very small amount of Fe (VI) to improve the removal rate of natural organics including humic acid (HA) (37). Additionally, Fe (VI) was found to be effective in promoting the oxidative transformation of pharmaceuticals such as fluoroquinolones (38–40), sulfonamides (41), β-lactams (42) and β-blockers (43). Since the removal of colistin has not been explored, we examined whether Fe (VI) could promote colistin removal from wastewater.

This study aimed to (i) assess the influence of Fe (VI) level, solution pH and reaction duration on the removal of colistin to identify optimum conditions; (ii) evaluate whether humic acid in wastewater would interfere with colistin removal by Fe (VI); (iii) measure the antibacterial activity of reaction mixtures against *E. coli, S. aureus*, and *B. subtilis* and (iv) determine the toxicity of colistin before and after Fe (VI) oxidation using a Microtox bioassay testing system. As far as we know, this is the first paper to study the removal of colistin from water using Fe (VI) treatment.

## Materials and Methods

### Reagents

Colistin sulfate (>66.7%) was obtained from China Institute of Veterinary Drug Control (Beijing, China). Peptone soy broth (TSB) and Mueller-Hinton broth (MHB) were purchased from Qingdao Hope (Qingdao, China). HPLC grade methanol, acetonitrile and formic acid purchased from Sigma-Aldrich (Munich, Germany). All other reagents were of analytical grade. Potassium ferrate solid [K_2_FeO_4_, Fe (VI)] of 99% purity was provided by Guangzhou Kexing Chemical (Guangzhou, China). Humic acid (HA) was obtained from Tianjin Berens Biotechnology (Tianjin, China). Ultrapure water was prepared using a Millipore Milli-Q system (Molsheim, France). Stock solutions of colistin (60 μM) were prepared by dissolving colistin sulfate in Milli Q water. Fe (VI) solution was prepared by adding solid Fe (VI) to 1 mM Na_2_B_4_O_7_/5 mM Na_2_HPO_4_ at pH 9.0.

### Removal of Colistin by Fe (VI) and Influence of Humic Acid

Batch oxidation tests were conducted in 50 mL conical flasks equipped with magnetic stirrers and contained 10 mL of Fe (VI) and 10 mL colistin solution at the initial concentration of 30 μM followed by pH adjustment. The mixtures were stirred at 500 rpm at 25.0 ± 0.2°C and 0.8 mL samples were removed and immediately passed through a 0.22 mm nylon membrane filter into a 2.0 mL vial containing 0.2 mL of sodium thiosulfate (100 mM). The sample was then applied to an Oasis HLB solid phase extraction (SPE) cartridge (Waters, Milford, MA, USA) prior to LC-MS/MS analysis.

The amount of Fe (VI) that optimized colistin removal was examined by adding Fe (VI) into a colistin solution to maintain the molar ratios of Fe (VI) and colistin at 5:1, 10:1, 20:1, 30:1, and 40:1. The pH of the mixture was then adjusted to 9.0, stirred for 60 min at ambient temperature and then quenched with sodium thiosulfate followed by being applied to Oasis HLB SPE cartridges as per above.

The effects of pH on Fe (VI)-mediated colistin removal was examined in a Fe (VI): colistin 30:1 solution and the pH of the mixture was adjusted to 5.0, 6.0, 7.0, 8.0, 9.0, and 10. The samples were stirred for 60 min and then quenched and applied to SPE cartridges as per above. These initial conditions were also used to determine the optimal reaction durations for the Fe (VI)-mediated colistin removal at pH 7.0. The samples were stirred and samples were taken at 30 s, 1, 2, 5, 10, 15, 30, 45, and 60 min, quenched with sodium thiosulfate and then cleaned up by Oasis HLB SPE as per above. HA interference in the Fe (VI)-mediated colistin removal was examined using 10 mL colistin solutions individually mixed with HA at 0, 1.0, 5.0, 15, 30, and 60 mg L^−1^ followed by the addition of 10 mL Fe (VI) to maintain a molar ratio of Fe (VI): colistin at 30:1. The pH of the mixture was then adjusted to 7.0. The samples were reacted and quenched and SPE cleaned up as per above.

All experiments were performed in triplicate and the mean ± SD values were calculated. Analysis of variance was conducted with SPSS 17.0 software (IBM, Chicago, IL, USA) to elucidate statistical differences between groups.

### Analytical Procedures

Colistin levels in solutions were determined using ultra-performance liquid chromatography-tandem mass spectrometry (UPLC-MS/MS). Chromatographic separation was performed with a TSKgel Amide-80 column (150 × 2.0 mm, 3.0 μm, Tosoh, Tokyo, Japan) in a column oven maintained at 40°C. Solvent A and B were 0.1% formic acid in water and acetonitrile, respectively. The flow rate was 0.6 mL min^−1^. Colistin was eluted with a linear gradient of 0–0.8 min, 5% A; 0.8–1.5 min, 5–80% A; 1.5–3.0 min, 80% A; 3.0–3.5 min, 80–5% A; 3.6–5 min, 5% A. The injection volume was 10 μL. An Acquity UPLC I-Class system coupled to a Xevo TQ-S mass spectrometer (UPLC-TQ-S/MS) was used for analyte determination (Waters). The conditions of instrumentation were set as follows: the electrospray ion source was used in positive ionization mode with multi-period multiple reaction monitoring (mpMRM) (Table 1). The operation conditions were as follows: source temperature, 120°C; capillary voltage, 3.0 kV; desolvation temperature, 400°C; desolvation gas flow rate, 950 L h^−1^; collision gas flow rate, 0.15 mL min^−1^, cone gas flow rate, 150 L h^−1^. The limit of detection (LOD) of colistin A and colistin B were 5 nM and limit of quantification (LOQ) of colistin A and colistin B were 15 nM.

**Table 1:** Multiple reaction monitoring settings for MS/MS analysis of colistin.

**Analytes**	**Pre-cursor ion (m/z)**	**Product ions (m/z)**	**Cone voltage (V)**	**Collision energy (eV)**
CSA^a^	390.7	379.1, 384.9^c^	2	12, 10
CSB^b^	385.9	374.4, 380.1 ^c^	4	12, 10

a *Colistin A*.

b *Colistin B*.

c *Quantification ions*.

### Antibacterial Activity Assays

Colistin degradation was determined using a solution of 30 μM colistin and Fe (VI) 900 μM under the optimal reaction conditions of pH 7.0, stirring at 500 rpm at 25.0°C for 60 min. Blank reaction solutions were prepared in the same way except that colistin was replaced by water. The bacterial strains *E. coli* ATCC 25922, *E. coli* K88, *S. aureus* and *B. subtilis* were used at 1 × 10^6^ CFU mL^−1^ and added to samples from the degradation assay employing broth microdilution assays to measure the antibacterial activities of the reaction solution. Briefly, colistin samples were 2-fold diluted in a 96-well microdilution plate in MHB. Inoculums (100 μL) prepared in MHB were added to each well and the plates were covered before incubated at 37°C for 16–20 h. The absorbance of the solutions at 600 nm was determined as a surrogate of cell density. The antibacterial activity of colistin at concentration of 30 μM was determined using the same method. Growth inhibition percentage was calculated from the corresponding absorbance value from the plate reader using Equation (1) (41), where the A_max_ represents the maximum value of absorbance which indicates no growth inhibition (0%). The inhibition growth I (%), therefore, varies from 0 to 100%.


(1) $$\begin{array}{r} I\left(\%\right)=\left(A_{\text{max}}-A\right)/A_{\text{max}}\times 100\% \end{array}$$

OriginPro 9.1.0 statistical software (Origin Lab, Northampton, MA, USA) was employed to calculate the 50% growth inhibition (EC_50_) on basis of the dose-response curve prepared by each reaction solution. To assess the antibacterial activity of each sample, a potency equivalent quotient (PEQ) value was calculated from Equation (2) (44) where Colistin_0_ and colistin_t_ represents colistin solution before and after Fe (VI) treatment.


(2) $$\begin{array}{r} PEQ=EC_{50}\left(colistin_{0}\right)/EC_{50}\left(colistin_{\text{t}}\right) \end{array}$$

### Ecotoxicity Assessment

The ISO standard (45) with a *Vibrio fischeri* luminescence bioassay was employed to examine the toxicity of colistin and its degradation products. In brief, *V. fischeri* were exposed to the samples before and after Fe (VI) treatment under the optimal reaction conditions for 15 min at 15 ± 0.5°C, followed by determination of their bioluminescence intensities by a GloMax Multi Detection System (Promega, Madison, WI, USA). Then we computed the relative inhibitory rate (IR%) according to Equation (3) (46), in which E_0_ represents the normalized bioluminescence intensities of colistin solution before Fe (VI) treatment and E represents that of colistin solution after Fe (VI) treatment.


(3) $$\begin{array}{r} Relative\;inhibitory\;rate\;\left(IR\%\right)\;=\;\left(E_{0}\;-\;E\right)/E_{0}\;\times \;100\% \end{array}$$

## Results and Discussion

The emergence of mobile colistin resistance genes has threatened the role of colistin as the last line of defense against multidrug-resistant Gram-negative bacteria. Nonetheless, colistin is still allowed to be used as a feed additive in many countries and it is therefore necessary to find an effective removal procedure before it enters the aquatic environment. In this study we examined colistin degradation by Fe (VI) oxidization.

### Optimization of the Conditions for Degradation of Colistin by Fe (VI)

#### Fe (VI): Colistin Molar Ratio Dependence

**Figure 1:**
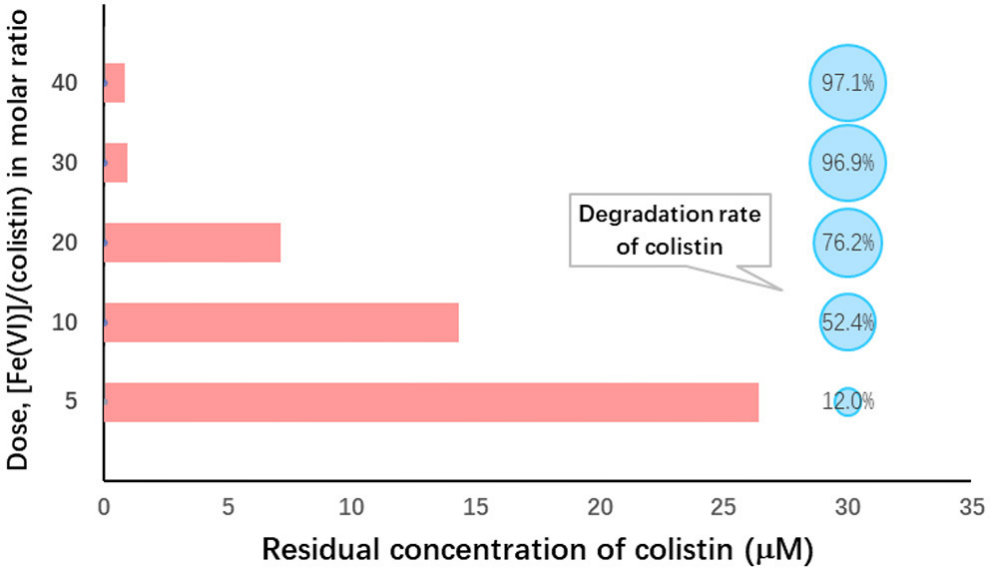
Ferrate (VI): colistin molar ratio dependence on colistin removal from solution. Initial colistin concentration, 30 μM; [Fe(VI)]/(colistin) molar ratio, 5–40; initial pH 9.0; temperature, 25°C; reaction duration, 60 min.

The initial dosage of Fe (VI) used is an important factor affecting its self-decomposition since it is proportional to its decomposition rate. In aqueous solution, this rate for Fe (VI) was previously determined to be 11% in solutions of 25 mM and complete decomposition occurred at levels of >30 mM within 60 min (47). We found that the molar ratio significantly affected colistin degradation (Figure 1). When the Fe (VI): colistin ration was increased from 5 to 30, colistin degradation rose from 8.3 to 96.9%. Degradation reached the level of statistical significance at molar ratios of 30 and 40 compared with the other ratios (*P* < 0.05; Table 2). The most likely reason for this was that at low K_2_FeO_4_ levels, the amount of Fe (VI) in solution available for reaction with colistin was insufficient and this was overcome at the higher K_2_FeO_4_ levels. Consistent with these interpretations, when the molar ratio increased from 30 to 40, colistin degradation increased slightly by only 0.8% and no significant difference between 30 and 40 was found (*P* > 0.05; Table 2). Results similar to these have been previously reported when Fe (VI) was employed to degrade bisphenol A (48). These reaction conditions provided sufficient Fe (VI) in solution to react with colistin and the further increase of potassium ferrate provided excess Fe (VI). The latter would however, accelerate its self-decomposition because elevated Fe (VI) concentrations are unstable (48) so that the effective concentration of Fe (VI) available for colistin degradation would be decreased. We therefore used a ratio of 30 for our remaining experiments since this level provided a high level of colistin degradation and was economical.

**Table 2:** Analysis of variance on degradation of colistin by different molar ratio of ferrate (VI) to colistin^a^.

**Molar ratio of ferrate (VI) to colistin**	***N***	**Subset of alpha** **=** **0.05 (Degradation rate,%)^b^**			
		**1**	**2**	**3**	**4**
5.00	3	8.3322			
10.00	3		52.3565		
20.00	3			76.2158	
30.00	3				96.9185
40.00	3				97.0968
Significance		1.000	1.000	1.000	0.946

a *Experimental conditions: initial colistin concentration = 30 μmol L^−1^; the molar ratio [Fe(VI)]/(colistin) = 5–40; initial pH = 9.0; temperature = 25°C; reaction duration = 60 min*.

b *Group mean values in a subset of the same type are displayed*.

#### Initial pH Dependence

The stability of ferrate is enhanced at higher pH (47) according to the following formula (49):


(4) $$\begin{array}{r} 4\;FeO_{4}^{2-}\;+\;10\;H_{2}O\;\to \;4Fe^{3+}\;+\;3O_{2}\;+\;12OH^{-} \end{array}$$

The production of OH^−^ from the reaction would lead to pH elevation that would also serve to inhibit Fe (VI) self-decomposition, thereby improving the stability of Fe (VI) in solution. In contrast, the presence of elevated H^+^ in solution as the pH falls would neutralize the generated OH^−^ and increase the reaction rate but also accelerate Fe (VI) decomposition. Therefore, Fe (VI) is more stable under alkaline conditions than under neutral and acidic conditions.

**Figure 2:**
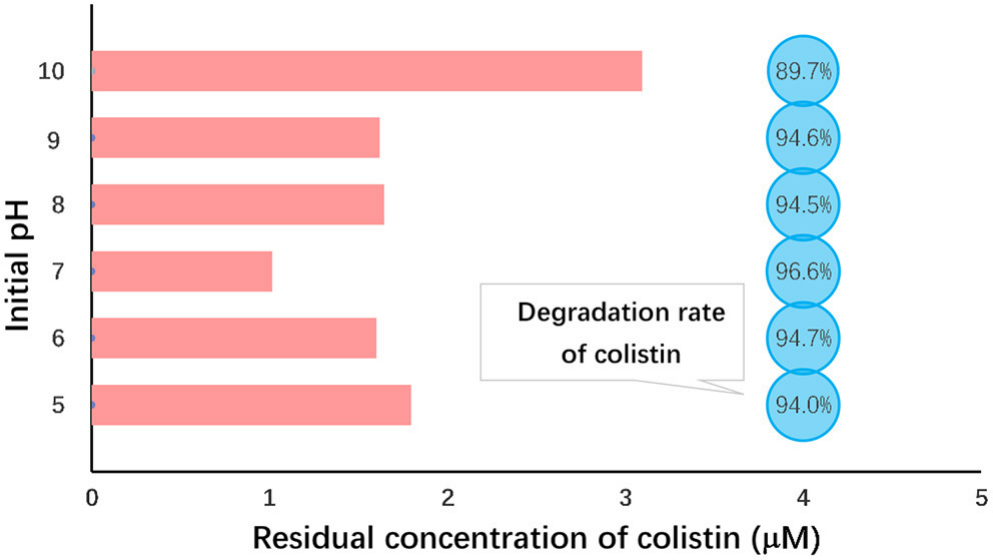
Initial pH dependence of colistin removal. Initial colistin concentration, 30 μM; [Fe (VI)]/(colistin) molar ration, 30; initial pH 5.0–10.0; temperature, 25°C; reaction duration, 60 min.

In this study, however, we found that the highest level of colistin degradation (96.6%) occurred under neutral conditions (pH 7.0) and the lowest (89.7%) was observed at pH 10 (Figure 2). Analysis of variance indicated that colistin degradation at pH 7 was significantly greater than those at all other pH values we examined (*P* < 0.05) and that at pH 10 was significantly lower than those at other pH values (*P* < 0.05; Table 3). Similar results were obtained when Fe (VI) was used to oxidize chloramphenicol (50). The initial pH of the solution most likely influences not only Fe (VI) stability which decreases as the pH declines, but also the oxidation capacity of Fe (VI) that decreases at elevated pH. Although Fe (VI) is stable under alkaline conditions, its low redox potential (0.7 V) restricts the reaction and colistin removal. Under acidic conditions, although Fe (VI) shows a high redox potential (2.2 V), its self-decomposition hindered the colistin reaction and Fe (VI) self-decay occurred rapidly within minutes at pH <6.0 (50, 51). Therefore, extremes of pH were not conducive to colistin removal and we selected pH 7.0 as the optimal initial pH for further study.

**Table 3:** Analysis of variance on degradation of colistin by ferrate (VI) at different pH values^a^.

**pH**	***N***	**Subset of alpha** **=** **0.05 (Degradation rate,%)^b^**		
		**1**	**2**	**3**
10.0	2	89.6829		
5.00	3		94.0103	
8.00	3		94.5154	
9.00	3		94.5998	
6.00	3		94.6629	
7.00	3			96.6167
Significance		1.000	0.293	1.000

a *Experimental conditions: initial colistin concentration = 30 μmol L^−1^; the molar ratio [Fe(VI)]/(colistin) = 30; initial pH = 5.0–10.0; temperature = 25°C; reaction duration = 60 min*.

b *Group mean values in a subset of the same type are displayed*.

#### Reaction Duration Dependence

**Figure 3:**
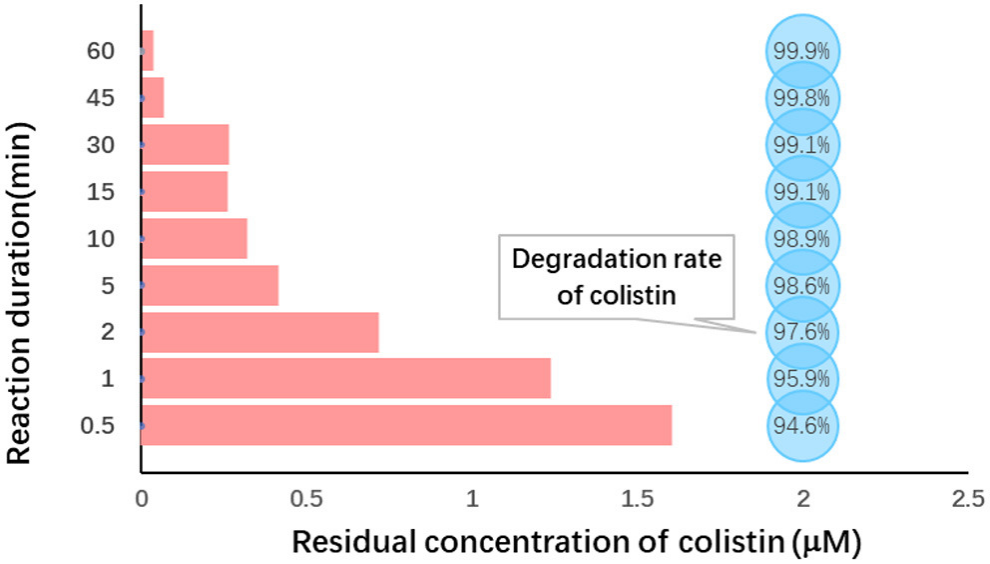
Reaction duration dependence of colistin removal. Initial colistin concentration, 30 μM; [F (VI)]/(colistin) molar ration, 30; initial pH, 7.0; temperature, 25°C, reaction duration, 0.5–60 min.

In order to achieve better degradation of colistin by Fe (VI), the effect of reaction duration was studied using the optimized level of Fe (VI) at pH 7.0. Colistin was degraded rapidly in the first 10 min and then the rate slowed (Figure 3). The degradation rate was significantly increased from 94.6 to 98.9% from 30 s to 10 min (*P* < 0.05) and remained at a plateau until 45 min and then increased slightly (Table 4). Due to the strong oxidizing power of Fe (VI), the majority of colistin (98.9%) was removed in the first 10 min. Following this, Fe (VI) consumption in the oxidation reaction as well as self-decomposition slowed the degradation rate. A time limit for the reaction was therefore set at 60 min to maximize colistin removal and was used in the remainder of this study.

**Table 4:** Analysis of variance on degradation of colistin by ferrate (VI) at different reaction duration^a^.

**Reaction duration (min)**	***N***	**Subset of alpha** **=** **0.05 (Degradation rate,%)^b^**					
		**1**	**2**	**3**	**4**	**5**	**6**
0.500	3	94.6452					
1.00	3		95.8749				
2.00	3			97.6093			
5.00	3				98.6102		
10.0	3					98.9248	
30.0	3					99.1104	
15.0	3					99.1210	
45.0	3						99.7714
60.0	3						99.8769
Significance		1.000	1.000	1.000	1.000	0.353	0.456

a *Experimental conditions: initial colistin concentration = 30 μmol L^−1^; the molar ratio [Fe(VI)]/(colistin) = 30; initial pH = 7.0; temperature = 25°C, reaction duration = 0.5–60 min*.

b *Group mean values in a subset of the same type are displayed*.

Colistin removal at 60 min was 99.9% and exceeded the level in the molar ratio and pH experiments where the maximum values were each 96.9 and 96.6%, respectively. When we examined the influence of Fe (VI) dosage on degradation, we set the initial pH at 9.0. In those experiments, the highest degradation rate was obtained at an Fe (VI): colistin molar ratio of 30 and pH 7.0 for 60 min. These slight discrepancies can be accounted for by differences in reaction batches of Fe (VI) due to (1) moisture, gas and atmospheric reducing substances that would interact with solid K_2_FeO_4_ and reduce the stability of Fe (VI) and (2) the rapid degradation of Fe (VI) under suboptimal conditions such that occur during pH adjustments that could alter the amount of active Fe (VI) present in the K_2_FeO_4_. However, since excess Fe (VI) was used in each reaction, trace amounts of colistin in the final reaction mixture were at the level of experimental error and not reaction condition. Therefore, Fe (VI) could readily and effectively remove colistin under the optimal conditions used in this study.

#### Interference of HA

Dissolved organic matter such as HA are common in wastewaters and natural water (52). We therefore examined whether HA at levels of 0–60 mg L^−1^ inhibited colistin removal by Fe (VI) under optimal conditions. We found that compared to the control, the presence of HA had no apparent impact on the degradation efficiency of colistin (*P* > 0.05; Table 5). Similar results were obtained in removal of polychlorinated diphenyl sulfides by Fe (VI) (52). The rapid reaction times for Fe (VI) and colistin most likely allowed this reaction to outcompete reactions with HA.

**Table 5:** Analysis of variance on degradation of colistin by ferrate (VI) in the presence of humic acid^a^.

**Concentration of humic acid (mg L^**−1**^)**	***N***	**Subset of alpha = 0.05 (Degradation rate,%)^b^ 1**
5.00	3	99.6582
0.00	3	99.6647
1.00	3	99.6786
15.0	3	99.6920
30.0	3	99.6925
60.0	3	99.7088
Significance		0.124

a *Experimental conditions: initial colistin concentration = 30 μmol L^−1^; the molar ratio [Fe(VI)]/(colistin) = 30; initial pH = 7.0; temperature = 25°C, reaction duration = 60 min*.

b *Group mean values in a subset of the same type are displayed*.

### Antibacterial Activity of Degradation Products

**Figure 4:**
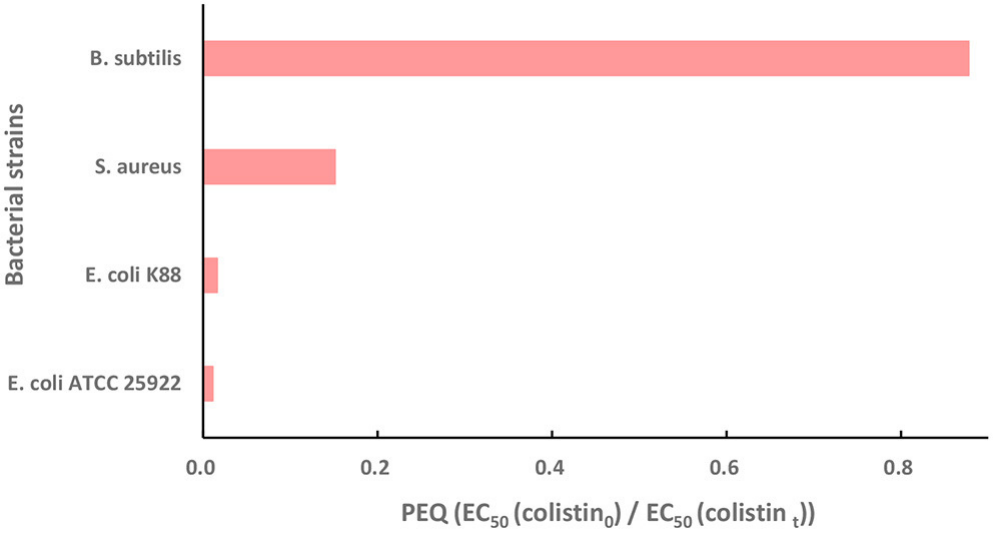
The potency equivalent quotient (PEQ) of colistin against *E. coli* ATCC 25922, wild type strain *E. coli* K88, *S. aureus* and *B. subtilis*. Initial colistin concentration, 30 μM; [Fe(VI)]/(colistin) molar ratio, 30; initial pH, 7.0; temperature, 25°C, reaction duration, 60 min. PEQ = EC_50_ (colistin_0_)/EC_50_ (colistin_t_) where colistin_0_ and colistin_t_ represent colistin before and after Fe (VI) treatment.

In order to find out if the colistin degradation products disturb the microecological balance in water and if they still pose selective pressure on generation and spread of *mcr*-1 in the environment, the antibacterial activities of colistin and its degradation products were determined with *E. coli, S. aureus*, and *B. subtilis* and we used PEQ analysis as a quantitative test of the dose-response curves. A PEQ value of 1 indicated that the EC_50_ of colistin before and after treatment were not different. A reduction in the antibacterial activity of colistin results in an increase in the EC_50_ of (colistin_t_) and a PEQ <1. Similarly, PEQ > 1 means that treatment enhances the antibacterial activity of colistin (53). All our experiments resulted in PEQ <1 (Figure 4), indicating decreases in antibacterial activity. The *E. coli* strains ATCC 25922 and K88 generated PEQ < 0.02 and *S. aureus* gave PEQ = 0.15, implying that the antibacterial activity of colistin against them was nearly eliminated by Fe (VI) oxidation. The *B. subtilis* PEQ was 0.88 and close to 1 and was the result of high EC_50_ (colistin_0_) and EC_50_ (colistin_t_) values. *B. subtilis* is a Gram-positive probiotic bacterium and is widely distributed in the environment. Since colistin is a cationic polypeptide that targets lipid A that is absent in Gram-positive bacteria (14, 54), the high value of EC_50_ (colistin_0_) against *B. subtilis* was expected. The value of PEQ close to 1 indicated that similar to colistin, the degradation products showed no obvious antibacterial activity to *B. subtilis* as well. However, it has been previously reported that colistin can destabilize the biofilm matrix structure even in species with intrinsic colistin resistance such as *S. aureus*, resulting in the release of planktonic cells that are more susceptible to antibiotics (55). In order to examine the change of antibacterial activity of colistin against both Gram-negative and Gram-positive bacteria, we included *E. coli*, the most common Gram-negative bacterium carrying plasmid-mediated resistance *mcr* genes as well as *S. aureus*, the representative Gram-positive bacteria in this study. The PEQ for *S. aureus* was higher than that for *E. coli* because the EC_50_ (colistin_0_) against *S. aureus* was ~10-fold greater than that against *E. coli*. Nonetheless, the EC_50_ (colistin_t_) against *E. coli* and *S. aureus* were similar. Fe (VI) has been proven effective in eliminating the activity of other antibiotics including trimethoprim (41), β-lactams (44) and fluoroquinolone (53). In conclusion, the colistin degradation products showed almost no antibacterial activity against representative Gram-negative and Gram-negative bacteria demonstrating that the method for colistin degradation was environmentally friendly. They would not affect the microecological balance in water and posed no selective pressure on generation and spread of *mcr-1* in the environment.

### Ecotoxicity Assessment

Colistin has significant side effects when used in humans and animals including nephrotoxicity that results in hematuria, proteinuria, urinary casts and increased blood urea and creatinine levels (54). In addition, this antibiotic is also neurotoxic with symptoms including paresthesia, peripheral neuropathy, muscle weakness and neuromuscular blockade resulting in respiratory paralysis (56). Colistin is also an exotoxin and is toxic to the earthworm *E. fetida* (17). However, there are no studies that assess the biological toxicity of colistin and its degradation products in water. We therefore wanted to determine whether colistin and its degradation products from Fe (VI) oxidation are non-toxic. For these experiments we measured alterations in the luminescence of *V. fischeri* with exposure to colistin and the degradation products. In order to remove the interference of Fe (VI) on the growth of *V. fischeri*, Fe (VI) activity at the end of the test periods were quenched using Na_2_S_2_O_3_ prior to bacterial exposure in the Microtox test.

Our results indicated that the IR% of colistin degradation products (2.99%) to *V. fischeri* was lower than that of colistin (7.38%), although these differences were not significant (*P* > 0.05). This was consistent with previous studies for the Fe (VI) oxidation of indomethacin (57), bisphenol A (48) and tetrabromobisphenol A (46). In the latter studies, the degradation products exhibited less toxicity to the *V. fischeri* bacteria compared to the parent drug. Our results also indicated that colistin and its degradation products were of low toxicity to the water environment because their IR% value were <30 (58).

One limitation of the current study is that we did not describe the degradation kinetics and identify the degradation products. Due to the large excess of Fe (VI) involved in the reactions, we found that >90% of colistin was degraded after reaction for 5 s (data not shown), indicating that most of degradation was completed nearly in an instant. The purpose of this study was to find an effective way to eliminate colistin in a short time and in turn, to reduce its risk of promoting the generation and spread of plasmid-mediated resistance *mcr* genes in the environment. The latter could cause failure as rescue treatments for human infections with multidrug-resistant bacilli rather than to compare subtle differences under different conditions. Therefore, we did not further study the degradation kinetics of colistin by Fe (VI) oxidation.

Since colistin sulfate is actually a mixture of colistin A and B as well as minor components, we were unable to identify all the components in the reaction mixtures using LC-MS/MS (data not shown). However, the final degradation products possessed minimal antibacterial activity against sensitive and non-sensitive bacteria and most had no toxicity to the luminescent bacteria. Hence, a failure to identify the products would not affect the application of Fe (VI) to remove colistin in practice under the optimal conditions proposed in this study.

## Conclusions

The degradation of colistin by Fe (VI) was investigated and we found that (1) colistin can be effectively degraded by Fe (VI) oxidation (99.9%) using an Fe (VI): colistin molar ratio of 30 with an initial colistin concentration of 30 μM at pH 7.0, 25°C for 60 min. (2) The presence of humic acid had no obvious impact on the removal of colistin by Fe (VI). (3) The products of colistin produced by Fe (VI) degradation possessed no antibacterial activity against *E. coli, S. aureus* and *B. subtilis*, indicating that Fe (VI) would be effective at reducing selective pressure of colistin on environmental bacteria for generation and spread of plasmid-mediated colistin resistance genes. (4) Both colistin and its degradation products in water were of low toxicity to *Vibrio fischeri*.

## Data Availability Statement

The original contributions presented in the study are included in the article/supplementary material, further inquiries can be directed to the corresponding author.

## Author Contributions

LW contributed to research, wrote the first draft of the manuscript, revision, read, and approved the submitted version. SL, XW, and BL contributed to data analysis, revision, read, and approved the submitted version. ZW contributed conception, revision, read, and approved the submitted version. All authors contributed to the article and approved the submitted version.

## Funding

This work was financially supported by National Natural Science Foundation of China (31760750, 32160853), Key Research and Development Project of Jiangxi Province (20171BBF60054), Science and Technology Projects of Jiangxi Provincial Education Department (GJJ180184), and Key Research and Development Project of Jiangxi Province (20161BBF60088).

## Conflict of Interest

The authors declare that the research was conducted in the absence of any commercial or financial relationships that could be construed as a potential conflict of interest.

## Publisher's Note

All claims expressed in this article are solely those of the authors and do not necessarily represent those of their affiliated organizations, or those of the publisher, the editors and the reviewers. Any product that may be evaluated in this article, or claim that may be made by its manufacturer, is not guaranteed or endorsed by the publisher.
